# Dosimetric comparison of intensity-modulated, conformal, and four-field pelvic radiotherapy boost plans for gynecologic cancer: a retrospective planning study

**DOI:** 10.1186/1748-717X-1-13

**Published:** 2006-05-04

**Authors:** Philip Chan, Inhwan Yeo, Gregory Perkins, Anthony Fyles, Michael Milosevic

**Affiliations:** 1 Department of Radiation Oncology, Princess Margaret Hospital-University Health Network, Toronto, Canada; 2 Department of Radiation Physics, Princess Margaret Hospital-University Health Network, Toronto, Canada; 3 Department of Radiation Oncology, University of Toronto, Toronto, Canada; 4 Institute of Medical Science, University of Toronto, Toronto, Canada

## Abstract

**Purpose:**

To evaluate intensity-modulated radiation therapy (IMRT) as an alternative to conformal radiotherapy (CRT) or 4-field box boost (4FB) in women with gynecologic malignancies who are unsuitable for brachytherapy for technical or medical reasons.

**Methods:**

Dosimetric and toxicity information was analyzed for 12 patients with cervical (8), endometrial (2) or vaginal (2) cancer previously treated with external beam pelvic radiotherapy and a CRT boost. Optimized IMRT boost treatment plans were then developed for each of the 12 patients and compared to CRT and 4FB plans. The plans were compared in terms of dose conformality and critical normal tissue avoidance.

**Results:**

The median planning target volume (PTV) was 151 cm^3 ^(range 58–512 cm^3^). The median overlap of the contoured rectum with the PTV was 15 (1–56) %, and 11 (4–35) % for the bladder. Two of the 12 patients, both with large PTVs and large overlap of the contoured rectum and PTV, developed grade 3 rectal bleeding. The dose conformity was significantly improved with IMRT over CRT and 4FB (p ≤ 0.001 for both). IMRT also yielded an overall improvement in the rectal and bladder dose-volume distributions relative to CRT and 4FB. The volume of rectum that received the highest doses (>66% of the prescription) was reduced by 22% (p < 0.001) with IMRT relative to 4FB, and the bladder volume was reduced by 19% (p < 0.001). This was at the expense of an increase in the volume of these organs receiving doses in the lowest range (<33%).

**Conclusion:**

These results indicate that IMRT can improve target coverage and reduce dose to critical structures in gynecologic patients receiving an external beam radiotherapy boost. This dosimetric advantage will be integrated with other patient and treatment-specific factors, particularly internal tumor movement during fractionated radiotherapy, in the context of a future image-guided radiation therapy study.

## Background

Intra-uterine brachytherapy following external beam radiotherapy is an integral component of the treatment of locally advanced cervix cancer. Patients who are unable to proceed with brachytherapy because of insufficient tumor regression during external beam radiotherapy, irregular pelvic anatomy or concurrent medical problems are at substantially higher risk of pelvic tumor recurrence [1,2]. It is possible that this largely reflects the intrinsically worse prognosis of patients with bulky disease at presentation, which regresses poorly in response to external radiotherapy. However, it may also reflect the lower total dose of radiotherapy that can safely be delivered to the tumor in the absence of brachytherapy. In support of the latter, several studies have suggested a dose-response relationship for cervix cancer [3-5].

Historically, patients who could not undergo brachytherapy received additional external beam radiotherapy delivered to the gross tumor alone in most radiation centres around the world, usually using a 4-field box technique (4FB). The dose of radiotherapy that could be delivered in this situation was limited by the tolerance of adjacent normal tissues, including bladder and particularly rectum. Modern techniques of precision radiation delivery like intensity-modulated radiation therapy (IMRT) allow the dose to be "sculpted" to the tumor volume while at the same time minimizing the dose to adjacent dose-limiting normal tissues [6,7]. This theoretically offers the opportunity to escalate the tumor dose with the expectation of improved local control. However, to achieve this goal, the IMRT boost needs to be delivered in an optimal manner with close attention to normal tissue dose-volume constraints and daily target localization.

The purpose of this study was to: 1) Quantify the potential advantage of an IMRT boost relative to conventional 4FB or conformal (CRT) techniques, and 2) Correlate rectal and bladder toxicity in patients treated using CRT with dose volume parameters, as a first step in establishing appropriate normal tissue dose constraints for this population.

## Methods

### Patient characteristics and treatment

Twelve patients with gynecologic cancer who received a CRT boost in the place of planned brachytherapy after large field pelvic radiotherapy (PRT) with or without concurrent chemotherapy were retrospectively identified. The characteristics of the patients are summarized in Table 1. All tumors were situated in the low central pelvis. There were eight cervical carcinomas, two vaginal vault carcinomas with previous hysterectomy for pre-invasive cervical disease, one endometrial carcinoma with vaginal vault recurrence, and one primary serous uterine carcinoma who had prior subtotal hysterectomy. In seven of the patients, brachytherapy was judged to be not feasible based on tumor location and residual disease bulk at the completion of PRT. In three cases, brachytherapy was attempted but technically was not feasible. Interstitial brachytherapy is not routinely practiced in this institution and hence was not an option for these patients. In the remaining two patients, brachytherapy was not attempted because of serious medical co-morbidity.

**Table 1:** Tumor characteristics and pelvic treatment summary for 12 patients with gynecologic tumors who received a CRT boost

Patient	Primary Site	FIGO Stage	Pelvic Technique	Posterior Pelvic Attenuator ^1^	PA RT	Pelvic Dose ^2^	Concurrent Chemotherapy ^3^
1	Vagina	3	POP	N	Y	50	N
2	Vagina	1	4FB	N	N	50	Y
3	Cervix	2B	4FB	Y	N	50	Y
4	Cervix	3B	POP	N	N	50	Y
5	Cervix	2B	4FB	N	N	45	Y
6	Cervix	2B	4FB	Y	N	50	Y
7	Cervix	1B	4FB	Y	N	45	N
8	Cervix	2B	4FB	Y	N	45	Y
9	Cervix	4A	4FB	N	N	45	N
10	Uterus	3A	4FB	N	N	45	Y
11	Uterus	Recurrent	4FB	N	N	45	N
12	Cervix	2B	4FB	N	N	45	Y

(1) 2 half-value posterior midline attenuator, 3 cm wide at mid-plane

(2) Delivered in 25 fractions over 5 weeks

(3) Cisplatinum 40 mg/m^2 ^weekly for 5 weeks

(FIGO) International Federation of Gynecology and Obstetrics

(PA RT) Para-aortic radiotherapy

(POP) Parallel opposed anterior and posterior fields

(4FB) Four-field box technique

Prior to CRT boost treatment, all patients received external beam pelvic radiotherapy using either a 4-field technique or opposed anterior and posterior beams if the primary tumor was too bulky to spare the posterior pelvis. The pelvic dose was 45–50 Gy in 1.8–2 Gy fractions over five weeks. A two half-value posterior midline attenuator was used in four patients to reduce the posterior rectal wall dose by approximately 20%. This is done routinely at our centre in patients receiving brachytherapy for cervix cancer to reduce the risk of serious late rectal morbidity [8]. One patient with gross pelvic lymphadenopathy also received treatment to para-aortic lymph nodes. Eight patients received concurrent intravenous chemotherapy with cisplatinum 40 mg/m^2 ^weekly. The median gap between PRT and CRT was 11 days (range 1–37 days) with the median overall treatment days of 63.5 days (range 54–92 days).

The tumor volumes and adjacent normal organs-at-risk (OAR) including bladder, rectum and bowel were defined for each patient by her individual treating physician, using information from a planning CT scan and pelvic MRI done after completing PRT specifically for these patients. The gross tumor volume (GTV) included the high T2 signal tissues identified using these scans. In general, they would be the residual tumor within the cervix with its invasion into the surrounding tissues or the residual vaginal vault tumor. The GTV was expanded 3-dimensionally(3D) uniformly by 1 cm and further adjustment by the individual oncologist based on the clinical history to define the clinical target volume (CTV), and then by a further 0.5 cm (3D) to define the planning target volume (PTV). This 0.5 cm PTV margin was chosen arbitrarily based on our departmental set-up error and did not account for organ motion error which is lacking in published literature at the time of this study. The median PTV was 151 cm^3^, with a range of 58–512 cm^3^. The outer aspect of the rectum was contoured from the level of the sciatic notch superiorly to the inferior aspect of the obturator foramen. The entire outer surface of the bladder was contoured. The median contoured rectal volume was 67 cm^3 ^(range 29–147 cm^3^), and the median bladder volume was 183 cm^3 ^(range 49–555 cm^3^). The PTV overlapped the contoured rectal and bladder volumes in most patients: on average 21% (range 1–56%) of the rectal volume was encompassed by the PTV, as was 13% (range 4–35%) of the bladder volume. The remaining small and large bowel was contoured from the level of the L5/S1 junction to the lower limit of the obturator foramen using the peritoneum as the surrogate. The median overlap with PTV was 0.98% (range 0–3%) reflecting on the low pelvic position of the PTV.

The CRT boost plans were optimized to deliver a uniform dose to the PTV while sparing critical normal tissues. Between five and eight coplanar 18-MV photon beams were used as chosen by the physicians and the dosimetrists at the time of original treatment as the optimal plan. The prescription dose was at the discretion of the treating physician and varied between 20 and 30 Gy (median 25.2 Gy) in 1.8–2 Gy daily fractions. Multi-leaf collimators (MLC) with 1 cm leaves were used to shape the fields to the beams-eye projections of the PTV. Table 2 summarizes the CRT as delivered to the patients.

**Table 2:** Summary of the CRT boost treatment

Structure	Parameter	All patients Median (Range)	Patient 4 Grade 3 Rectal Bleeding	Patient 10 Grade 3 Rectal Bleeding
Tumor	Dose (Gy)	25.2 (20–30)	20	30
	PTV (cm^3^)	151 (58–512)	512	393
	CN	0.58 (0.34–0.78)	0.74	0.75

Rectum	Volume^1 ^(cm^3^)	67 (29–147)	61	73
	Overlap with PTV^2 ^(%)	15 (1–56)	41	46
	V_50 _(cm^3^)	57 (22–128)	60	63
	V_70 _(cm^3^)	45 (16–113)	58	53

Bladder	Volume^1 ^(cm^3^)	183 (49–555)	148	325
	Overlap with PTV^2 ^(%)	11 (4–35)	35	20
	V_50% _(cm^3^)	125 (32–187)	148	161
	V_70% _(cm^3^)	79 (23–145)	145	106

(1) Rectal and bladder contoured volume before expansion to form the planning risk volume (PRV)

(2) Percentage of the rectum or bladder volume within the PTV

(V_50%_)The volume receiving ≥ 50% of the prescribed dose

(V_70%_) The volume receiving ≥ 70% of the prescribed dose

(PTV) Planning target volume

(CN) Conformation Number.

Patients were followed at 3 monthly intervals for the first two years after completing radiotherapy, and at six monthly intervals thereafter. At each visit, the clinical history was updated and a physical examination, including pelvic examination, was performed. Laboratory and imaging tests were obtained as required based on clinical findings but an MRI scan was done routinely for these patients at 6 months post treatment. Late radiation complications were scored using the RTOG scale for both gastrointestinal and genitourinary system. The median follow-up was 1.9 years, with a range of 5 months to 3.3 years. This retrospective study was approved by the Research Ethics Board of the University Health Network and Princess Margaret Hospital.

### Comparison of IMRT with CRT and 4FB

Optimized IMRT boost treatment plans were developed for each of the 12 patients to determine the benefit of IMRT in this clinical setting. The IMRT plans for each patient were compared to the CRT plan and to a 4FB technique, which is historically how patients ineligible for brachytherapy have been treated in many centres in particularly those not familiar with interstitial techniques. The target and normal tissue volumes, as delineated by the treating physician for each patient, were unaltered for the purpose of this planning exercise. Rectal and bladder planning risk volumes (PRVs) for IMRT were defined by adding a uniform margin of 0.5 and 1 cm respectively to the contoured organ volumes to assist IMRT dose optimization.

The 4FB plans were generated by adding a uniform 1 cm margin to the PTV to account for beam penumbra. MLC corner shielding was used with a beam energy of 18 MV for each of the 4-beams.

### IMRT planning

**Figure 1:**
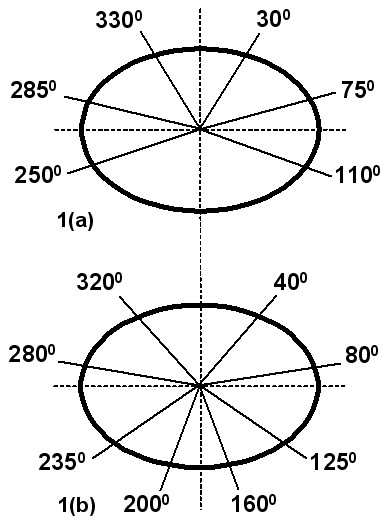
Beam arrangements for the six (a) and eight (b) field IMRT plans.

Two IMRT boost plans were developed for each patient using six or eight coplanar beams as shown in Figure 1. The beam arrangements were chosen to be symmetrical about the PTV and to avoid treating directly through the rectum or bladder, which are the major dose limiting organs. Inverse planning was performed to optimize PTV dose uniformity (-5 to +7%, ICRU62), and secondarily to minimize dose to the rectum and bladder [9]. PTV coverage was not compromised as a result of overlap with the rectal and bladder PRVs. The dose-volume constraints for the portions of the rectum and bladder outside of the PTV were set so that 30% of the PRV received less than 66% of the prescribed dose, and 70% of the PRV received less than 33% of the dose. The IMRT plans were based on a sliding window method using 6 MV photons to minimize neutron contamination [10]. All treatment planning was done with CadPlan/Helios v6.2.7. (Varian Medical Systems, Inc. Palo Alto, CA).

### Treatment plan evaluation

The IMRT treatment plans were compared to the CRT and 4FB plans with respect to PTV coverage and avoidance of adjacent critical normal tissues. To facilitate this comparison of this planning study, a uniform dose of 25.2 Gy was re-prescribed at the isocenter (the median of the doses actually delivered to the 12 patients). Cumulative DVHs were generated for the PTV, contoured rectal volume, and contoured bladder volume.

The Conformation Number (CN) as described by van't Riet *et al*. [11] was used to assess the PTV plan conformity. Although Feuvret *et al*. [12] concluded in their review that the future of conformity indices in everyday practice remains unclear, we have selected this index as the best available method in comparing both target coverage as well as normal tissue avoidance in our data. The CN, in the context of this analysis, was defined as the product of the proportion of the PTV encompassed by the 95% isodose volume, and the proportion of the 95% isodose volume accounted for by the PTV:



The first term provides an indication of how well the PTV is covered by the 95% isodose volume. This was optimized during treatment planning, and was greater than 0.95 for all of the IMRT and CRT plans. The second term indicates the extent to which the 95% isodose volume extends beyond the PTV, potentially encompassing adjacent OARs. Overall, the CN has a range of possible values from 0, indicating complete geographic miss of the PTV, to 1, indicating perfect conformality of the 95% isodose volume to the PTV.

**Figure 2:**
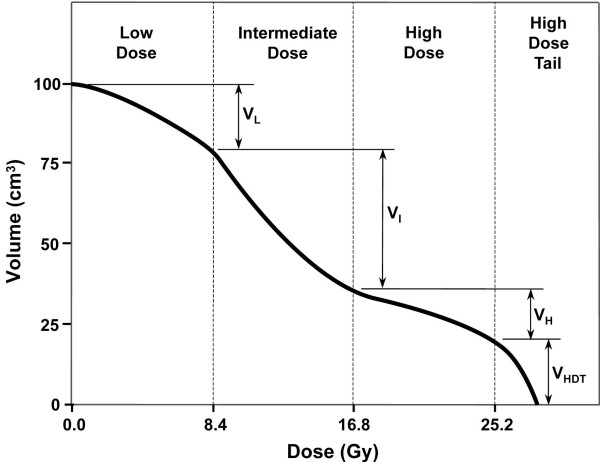
Hypothetical cumulative DVH for rectum or bladder. Each DVH was divided into low dose (0–33% of the prescription), intermediate dose (34–66%), high dose (67–100%), and "high-dose tail" (>100%) regions. The volume of tissue receiving doses in each of these ranges (V_L_, V_I_, V_H_, V_HDT_) was calculated.

Normal tissue avoidance was evaluated using the cumulative DVHs for rectum and bladder. Each DVH was divided into low, intermediate, high and "high-dose tail" regions. The tissue volumes receiving doses in these four ranges (V_L_, V_I_, V_H_, V_HDT_) were derived from the DVH data, as illustrated in Figure 2. V_L _represented those volumes treated up to 33% of the prescribed dose, while V_I_, V_H_, and V_HDT _represented the volumes received between 34 to 66%, 67 to 100% and >100% of the prescribed dose, respectively. V_H_+V_HDT _is equivalent to the volume of tissue receiving greater than 66% of the prescribed dose (V_66%_), and V_HDT _to the volume receiving greater than 100% of the prescription dose (V_100%_). This was divided into thirds for ease of comparison due to the heterogeneity of the original prescribed doses.

## Results

### Clinical outcome after conformal treatment

Nine of 12 patients had complete clinical regression of disease 12 months after completing CRT. Two patients had a partial response, and the remaining patient had progressive pelvic disease and developed distant metastasis. One of the nine patients who regressed completely recurred within the high-dose volume 1.8 years later.

Two patients (numbers 4 and 10) developed grade 3 rectal toxicity with bleeding necessitating intervention. In one (patient 4) the pelvic tumor was controlled, while the second (patient 10) developed central pelvic recurrence 3 months before the onset of rectal bleeding. Both underwent colonoscopy to establish the diagnosis of late radiation proctitis. As summarized in Tables 1 and 2, both received PRT with a 4-field technique and concurrent chemotherapy. The posterior attenuator was not used in either case at the discretion of the treating physician. Both had bulky residual disease at the completion of PRT: the PTVs for these two patients (512 and 393 cm^3^) were the largest in the cohort. In addition, the percentage of the rectal volume that overlapped the PTV was near the high end of the range in both cases (41% and 46%): only one patient who did not manifest late rectal toxicity had greater overlap with the PTV (56%). There was no genitourinary toxicity greater than grade 2 observed.

### Comparison of IMRT with CRT and 4FB

Dose conformality and normal tissue avoidance were used to evaluate the two IMRT beam arrangements. As shown in Table 3, the CN's for the 6 and 8-field IMRT plans did not differ significantly, nor did the CN's for the CRT and 4FB plans. However, the use of IMRT (6 or 8-fields) significantly improved the conformality relative to CRT or 4FB treatment (p ≤ 0.001). The 95% isodose line encompassed 95% or more of the PTV in all of the IMRT and CRT plans, and in most of the 4FB plans. Therefore, the improvement in CN with IMRT resulted mainly from improved conformality of the 95% isodose to the PTV (second term in Equation 1), with exclusion of adjacent normal tissues.

**Table 3:** PTV dose conformality for the IMRT, CRT and 4FB plans in 12 patients

Technique	CN Median	Range CN	p
IMRT – 6 field	0.75	0.61–0.87	0.001
IMRT – 8 field	0.75	0.60–0.84	<0.001
CRT	0.6	0.36–0.74	NS
4FB	0.59	0.53–0.62	

(4FB) Four field box technique

(CN)	Conformation number. A value of zero indicates complete geographic miss of the PTV. A value of 1 indicates perfect conformality of the 95% isodose volume to the PTV.

(CRT) Conformal radiotherapy

(IMRT) Intensity-modulated radiotherapy

(NS) Not significant

(p) p-value by paired t-test relative to 4FB

Table 4 summarizes the DVHs for the dose-limiting normal tissues: rectum and bladder. For each IMRT and CRT plan, ratios were calculated for the volumes of tissue receiving doses in each of the four previously defined ranges (V_L_, V_I_, V_H_, V_HDT_) to the corresponding 4FB volumes in the same patient. A ratio >1 indicates a larger volume of normal tissue receiving doses in a particular range, while a ratio <1 indicates relative sparing of normal tissue in that dose range. This allows evaluation of the relative benefits of IMRT and CRT in individual patients relative to 4FB treatment, as well as in the entire cohort overall.

**Table 4:** Normal tissue avoidance for the IMRT and CRT plans in 12 patients, Ratio relative to a 4FB technique

Technique	OAR	Median V_L _(Range)	Median V_I _(Range)	Median V_H _(Range)	Median V_HDT _(Range)	Median V_H_+V_HDT _(Range)
IMRT – 6 field	Rectum	2.6 (1.1–168)	1.2 (0.4–16.4)	0.7 (0.4–1.5)	2.1 (0–134)	0.78^1,2 ^(0.48–0.98)
	Bladder	10.7 (1.6–314)	1.0 (0.4–5.1)	0.7 (0.5–0.9)	2.9 (1.0–569)	0.81^1,2 ^(0.65–0.98)
IMRT – 8 field	Rectum	1.8 (0.1–166)	1.1 (0.6–9.5)	0.9 (0.6–1.5)	0.9 (0.1–208)	0.90^1,2 ^(0.61–1.06)
	Bladder	7.1 (1.7–490)	1.0 (0.6–8.5)	0.8 (0.6–0.9)	0.8 (0–17)	0.78^1,2 ^(0.67–0.88)
CRT	Rectum	1.1 (0–71)	1.0 (0.4–12)	1.0 (0.3–1.3)	1.0 (0–1.8)	1.02 (0.61–1.3)
	Bladder	7.8 (1.9–336)	0.55 (0.3–0.9)	0.87 (0.4–1.4)	1.1 (0–403)	0.96 (0.85–1.4)

(1) IMRT vs. 4FB, p ≤ 0.001 by t-test

(2) IMRT vs. CRT, p ≤ 0.05

(CRT) Conformal radiotherapy

(IMRT) Intensity-modulated radiotherapy

(4FB) Four field box technique

(V_L_)Volume of tissue receiving ≤ 33% of the prescription dose

(V_I_)Volume of tissue receiving 34–66% of the prescription dose

(V_H_)Volume of tissue receiving 67–100% of the prescription dose

(V_HDT_)Volume of tissue receiving >100% of the prescription dose (V_100%_)

(V_H_+V_HDT_)Volume of tissue receiving >66% of the prescribed dose (V_66%_)

As shown in Table 4, both 6-field and 8-field IMRT significantly reduced the volume of rectum and bladder receiving high doses >66% of the prescribed dose (V_H _+V_HDT_) relative to 4FB treatment. Six-field IMRT produced a 22% reduction in the volume of rectum receiving the highest doses and 19% reduction in the high-dose bladder volume, compared to reductions of 10% and 22% respectively for 8-field IMRT. There was a trend towards greater high-dose sparing of the rectum with 6-field IMRT. The volume of rectum receiving doses in the highest range (V_H _+V_HDT_) was reduced with 6-field IMRT relative to a 4FB technique in all 12 patients, although the relative reduction was <5% in three of the patients. These three all had large PTVs (191–512 cm^3^) and large overlap of the rectum with the PTV (28–56% of the contoured rectal volume). Six-field IMRT produced a median reduction of 9.2 cm^3 ^in the absolute volume of rectum receiving the highest doses, with values in individual patients ranging from 0.7 cm^3 ^to 26 cm^3^. The two IMRT plans resulted in equivalent high-dose sparing (V_H _+V_HDT_) of bladder compared to CRT or 4FB treatment.

Shown in Table 4, the high-dose sparing with IMRT was mostly due to a reduction in the volume of tissue receiving doses between 67% and 100% of the prescribed dose (V_H_). Both 6-field and 8-field IMRT resulted in a prominent "high-dose tail" on the rectal and bladder dose-volume histogram (Figure 2) in some patients, corresponding to an increased volume of normal tissue receiving doses above the prescribed dose (V_HDT_). This effect was greater with 6-field than with 8-field treatment, where it tended to offset the dosimetric advantage of reduced V_H_. The median increase in rectal V_HDT _was 110% with 6-field IMRT relative to 4FB treatment, compared to a 10% reduction in median V_HDT _with 8-field IMRT. The large relative increases in V_HDT _seen in some patients (>100-fold) mainly reflected very small volumes of rectum receiving doses in this range with 4FB treatment. The median absolute rectal V_HDT _was 3.2 cm^3 ^(range 0.5–41 cm^3^) for 6-field IMRT, and 2.6 cm^3 ^(range 0.5–12.7 cm^3^) for 8-field treatment. For bladder, the corresponding numbers were 8.8 cm^3 ^(range 0.3–21 cm^3^), and 3.7 cm^3 ^(range 0.1–7.9 cm^3^).

The volume of rectum and bladder receiving doses from 0 to 33% of the prescribed dose (V_L_) also increased in most cases with either 6-field or 8-field IMRT relative to a 4FB technique. The rectal V_L _increased by a factor of 2.6 (range 1.1–168) with 6-field IMRT, and by a factor of 1.8 (range 0.1–166) with 8-field treatment. Again, the large relative increases that were seen in some patients largely reflected the fact that very small volumes of the rectum and bladder received low doses in this range with the 4FB technique. The median absolute rectal V_L _values were 7.9 cm^3^and 3.4 cm^3 ^respectively with 6- and 8-field IMRT. Similar trends were observed for the bladder, with corresponding median V_L _values of 28.3 cm^3 ^and 10.5 cm^3^.

## Discussion

Ideally, patients with cervix cancer who are candidates for curative treatment with radiotherapy should receive a combination of external beam treatment and brachytherapy [13,14]. However, in our practice, between 5% and 10% of patients are not candidates for brachytherapy because of either patient or tumor-specific limitations. These patients are potential candidates for additional external beam radiation to the primary tumor, and might benefit from dose escalation with IMRT. In this study, we identified a cohort of 12 patients with gynecologic tumors who were unsuitable for brachytherapy and received a CRT boost in the pre-IMRT era. Tumor control and toxicity for these patients were correlated with dose-volume parameters, and the cases were re-planned to assess the potential benefit of IMRT. The results demonstrate significant rectal and bladder dose sparing in most patients' plans. This is the first comparison report of standard fractionation 4FB, CRT, and IMRT in a cohort of this important but yet relatively small group of patients who are unable to undergo brachytherapy, and whose outcome may be compromised as a result. There is evidence in the literature that IMRT will improve target coverage and improve normal tissues sparing for whole pelvic IMRT [6,7,15,16] as well as a recent study by van de Bunt *et al*. [17] that demonstrated repeated IMRT planning as the tumor shrinks is also advantageous over conventional and CRT planning for whole pelvic IMRT. One recently published report by Mollà *et al*[18] showed that it is feasible to use external beam stereotactic radiotherapy using dynamic-arc or IMRT method as a boost for this population of patients. However, unlike this current paper it did not quantify the degree of improvement that the precision method can have over a conventional external beam boost where it is more accessible by all radiation centres around the world. However, we recognize that to properly identify dose limitations and toxicity of surrounding normal tissues, summation of the DVH of PRT and CRT is needed. However this is beyond the scope of this retrospective study where variations existed within the cohort for both the prescribed dose of PRT and CRT as well as the deformation of the volumes between these 2 phases of treatment.

### Conformal boost toxicity

As outlined in Table 1 and 2, the treatment received by these 12 patients was variable in terms of external beam volume, the use of a posterior attenuator (designed to limit the rectal external beam dose in the region of highest brachytherapy dose), the use of concurrent cisplatinum chemotherapy during external radiation, and the prescribed CRT boost dose and fractionation. These factors, which all might influence late toxicity, together with the small number of patients and the relatively short follow-up particularly for genitourinary side effects [19,20], prevent us from drawing firm conclusions about the relationship between radiotherapy dose-volume parameters and toxicity. The late complication rate of 17% following CRT boost treatment that was observed in this series may be slightly higher than with brachytherapy. By comparison, we identified a 7.5% rate of grade 3 or 4 complications in 166 patients with cervix cancer who received either LDR or PDR brachytherapy boost following external radiotherapy [8]. The two patients in the current series that developed late rectal bleeding both had large PTVs and large overlap between the contoured rectal volumes and the PTV. IMRT, which reduced the volume of rectum receiving doses in the highest range in most cases, improved the dose distribution in one of these patients relative to either CRT or a 4FB approach (V_H_+V_HDT _ratios of 0.71 and 0.77 respectively for 6-field IMRT vs. CRT and 4FB, patient 10) but was of little benefit in the other (V_H_+V_HDT _ratios of 0.94 and 0.96 respectively, patient 4). Overall, 6-field IMRT resulted in potentially significant high-dose rectal and bladder sparing relative to CRT or 4FB treatment in nine of 12 patients (V_H_+V_HDT _reduction >10%). This implies that, while IMRT has the potential to benefit most patients in this clinical setting, it may be of less value in those with bulky residual disease in close proximity to rectum or bladder at the completion of large-field pelvic radiotherapy.

Our study provides preliminary information about the relationship between dose-volume parameters and late radiation complications following external beam boost treatment for patients with cervix cancer. However, more accurate and comprehensive data are essential if the potential of IMRT to expand the therapeutic ratio is to be maximized in this cohort [21]. Useful data are beginning to emerge from studies of other pelvic malignancies treated with high-dose radiotherapy, notably prostate cancer [22-25]. In addition, detailed dosimetric studies of patients receiving brachytherapy for cervix cancer, using MR-compatible radiation applicators and post-insertion imaging to generate accurate DVHs for tumor and the critical OARs [26-30], will provide valuable information in this regard.

### Comparison of IMRT with CRT and 4FB

This study compares IMRT, CRT and 4FB treatment plans in patients with gynecologic tumors unable to undergo intracavitary brachytherapy boost, with respect to both tumor dose conformality and critical normal tissue avoidance. The CN provides an indication of how well the 95% isodose "hugs" the PTV, and may be superior to the ICRU Conformity Index [9] to the extent that it also accounts for the tissue volume outside of the PTV that receives 95% of the prescription dose or higher [11]. Perfect conformality will yield a CN of 1.0, while plans where the prescription isodose extends beyond the PTV will yield values <1. IMRT produced approximately a 25% (p < 0.001) improvement in the CN relative to either the CRT or 4FB techniques (Table 4), which is of potential clinical significance. There was no difference in the conformality of the CRT and 4FB plans. This is not surprising given that conformal corner shielding with multi-leaf collimators was used in the 4FB plans, so that these two techniques converge in this respect.

Normal tissue avoidance was assessed using cumulative DVH's for rectum and bladder, which were divided into four regions to provide an indication of the volume of tissue receiving doses in the low, intermediate, high and "high-dose tail" ranges (Figure 2). In most of the patients, IMRT reduced the volume of rectum and bladder receiving doses between 67% and 100% of the prescription (V_H_), although this was partially offset by an increased volume of normal tissue receiving doses >100% (V_HDT_). When these two volumes were combined (V_H _+ V_HDT_), a net dosimetric advantage of IMRT persisted in most patients: the volume of rectum that received the highest doses was reduced by 22% with 6-field IMRT and the volume of bladder by 19%. This was at the expense of an increase in the volume of these organs receiving doses in the lowest range (V_L_). Table 4 indicates substantial relative increases in rectal and bladder V_L _in some patients. However, the absolute tissue volume receiving doses in the lowest range was usually small. For example, the median rectal V_L _was 10.6% (range 1–70%) of the total rectal volume with 6-field IMRT, 7.3% (range 1–52%) with 8-field IMRT, and 2.7% (range 0–48%) with 4FB radiation. Whether or not the high-dose sparing advantage of IMRT is offset in some patients because of an increase in the volume of normal tissue receiving lower dose resulting higher risk of secondary malignancies [31] will need to be addressed in future larger studies where dose-volume parameters and outcome are carefully correlated.

An almost infinite number of beam arrangements are theoretically possible when developing IMRT treatment techniques. We chose to use a coplanar technique and to prevent beams from entering or exiting the patient through the bladder and rectum, which are the major dose-limiting normal tissues. Therefore, the beams were arranged laterally and as symmetrically as possible about the patient and PTV. Two beam arrangements were examined, one with six fields and the other with eight fields. Both yielded significant improvement in the CN relative to CRT or 4FB treatment, and there was no difference between them in this respect. The 6-field IMRT technique produced greater rectal sparing in the dose range between 67% and 100% of the prescribed dose (V_H_) compared to 8-field IMRT, but a more prominent high-dose tail (V_HDT_). The net effect when these volumes were combined (V_H_+V_HDT_) suggested a benefit overall for 6-field treatment, as shown in Table 4. With respect to the bladder, 8-field IMRT yielded greater reduction in both V_H _and V_HDT _relative to 4 FB treatment, and may therefore be preferred over the 6-field technique. The relative advantages and disadvantages of these two beam arrangements will likely vary from patient to patient, and will need to be considered on an individual basis.

We compared idealized IMRT, CRT and 4FB treatment plans in patients with gynecologic tumors unable to undergo brachytherapy boost. It demonstrated a dosimetric advantage to IMRT that should allow dose escalation and/or a reduction in toxicity relative to other external beam approaches. However, it did not consider patient and treatment-related factors that might influence tumor control or toxicity independent of the boost technique, such as daily setup variability, internal tumor and organ movement, tumor regression during radiotherapy, and the characteristics of the initial large-field pelvic treatment [21]. Preliminary data from our centre have suggested significant movement of the PTV between radiation fractions in patients with cervix cancer [32], which might increase the risk of geographic miss given the higher dose gradients associated with IMRT. Daily on-line imaging with compensation for inter-fractional PTV movement may be necessary to overcome this problem, and might enhance the dosimetric advantage of IMRT even further by allowing narrower margins around the CTV. This supports the need for image-guided radiation therapy (IGRT). The use of concurrent chemotherapy during the initial phase of pelvic treatment and extended-field radiotherapy to encompass para-aortic lymph nodes may both increase the risk of radiation complications [33-36], which underscore the importance of integrating all aspects of treatment in individual patients so as to maximize outcome.

## Conclusion

This study demonstrates that conformal external beam radiotherapy can safely be delivered as a boost to the residual tumor in patients with gynecologic cancers who are unsuitable for brachytherapy. IMRT produces improved PTV conformality and high-dose sparing of rectum and bladder relative to CRT or 4FB treatment, and should allow dose escalation with the expectation of improved outcome. This information will need to be carefully integrated with other patient and treatment-specific factors, particularly documentation of internal tumor movement during fractionated radiotherapy in terms of online IGRT, to assure optimal patient outcome.

## Competing interests

The author(s) declare that they have no competing interests.

## Authors' contributions

PC conceived of the study, participated in the design, carried out the collection, analysis, interpretation of the data and drafted the manuscript. IY participated in the design and carried out the collection and analysis of the data and helped drafted the manuscript. GP participated in the conception and design of the study as well as collection and analysis of the data. AF participated in the conception, analysis and interpretation of the data as well as drafting of the manuscript. MM participated in the conception and design of the study as well as the analysis, interpretation of the data and drafting the manuscript. All authors read and approved the final manuscript.

**Figure 3:**
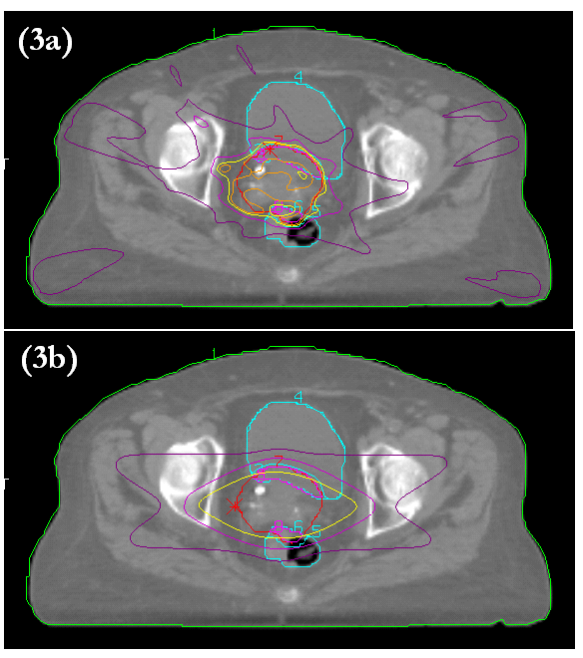
Representative axial isodose distributions for six-field IMRT (a) and conformal (b) treatment plans. The red line is the PTV. The yellow line is the 95% isodose.
